# When and why voice to higher-up? Declaring the psychological mechanisms of subordinate’s voice behavior in the public sector

**DOI:** 10.1371/journal.pone.0285104

**Published:** 2023-08-18

**Authors:** Khawaja Asif Tasneem, Zonghe Zhang, Sirui Sun

**Affiliations:** 1 School of International and Public Affairs, Shanghai Jiaotong University, Shanghai, China; 2 Law School of Shanghai University of International Business and Economics, Shanghai, China; 3 School of Humanities, Donghua University, Shanghai, China; 4 Shanghai Innovation Policy Evaluation Center, Shanghai, China; Wroclaw University of Economics and Business: Uniwersytet Ekonomiczny we Wroclawiu, POLAND

## Abstract

Based on the leadership-member exchange perspective, this study proposes that subordinates are more likely to express their voice in a leader-supported work environment, and this relationship is stronger when they have close social ties with their supervisor. In the case of subordinates supported by supervisors, public service motivation serves as a psychological mechanism to promote them to express voice behavior. This study also explains the boundary effect of the supervisor-subordinate’s guanxi perspective in affecting supervisor support and subordinate’s voice behavior. A longitudinal survey of 136 front-line public officers has been conducted to check this theoretical model in China, and their data verified the moderated-mediation model results. Implications for management theory and practice are discussed.

## 1. Introduction

Voice behavior is not only "communication" or "information sharing" that is universal, but also the input behavior of individuals who point out opportunities and ways to improve the organization. When public sectors created an environment, public employees could be encouraged to participate actively and share information, maximizing the human capital value in return. Against this background, many researchers investigated the motivation topics of individuals’ voice behaviors. Although these management studies are in their fashion, research on front line public officers’ voice behaviors is still in its infancy. Voice behavior also needs to be encouraged in the public sector. For example, voice behaviors warmly welcome some special jobs such as front-line administrative law enforcement officers. It is worth encouraging public law enforcement institutions because it created a bridge for public officers to challenge the authority for improper disposal of illegal and provided suggestions such as improving law enforcement procedures.

Organizations deficient in voice are problematic [1]. However, voice behavior is a discretionary proactive behavior, and supervisors cannot force employees to voice their, nor can they punish them for not voicing [2]. So, this study will explore how and when the voice behavior fire would be igniting among these public officers, and declare the psychological mechanism of subordinate voice behavior to their higher-ups. Existing research about the motivation mechanism of voice behavior was mostly conducted in for-profit organizations, i.e., companies. Then, what’s special for the employees of the public sector? Without a concrete measure (e.g., profit) to indicate the success of the organizations, how the employees’ working conditions, supervisor support, working motivation, etc. may differ from those in companies to serve as a motivation tool for public employees to express voice? Therefore, in this study we will try to discover the differences in the mechanisms that generate the suggestion behavior of frontline public sector employees and try to find the psychological mechanisms that motivate them to be brave and proactive in expressing suggestions at work, starting from the leadership relationship and the psychological mechanisms of individuals.

According to the leader-member exchange theory(LMX theory), superior-subordinate relationships would be important as a social connection in influencing subordinates’ behavioral performance [3]. Therefore, a subordinate’s voice behavior is equally susceptible to the influence of superior-subordinate relationships. For example, research has revealed that the supportive environment created by supervisors makes subordinates more likely to engage in proactive behavior as well as improve creativity (Liu et al., 2010) [4]. However, the support given by the supervisor in the work environment only answers the question of why the subordinate is willing to speak up, but the personal relationship between the supervisor and the subordinate needs to be considered as to whom the subordinate would prefer to speak up a ton this study, we argue that the closeness of personal relationships between superiors and subordinates leads individuals to decide to whom they should address their words of advice. For the front-line public employees in this study, the innovative suggestions they offer in response to their work are often inhibiting suggestions after problems have been identified, a risky initiative that challenges authority, and therefore the risky initiative is more likely to be listened to and heard by those they know personally and are close to as supervisors. On the one hand, because they have more contacts, both work and personal, and on the other hand because they trust that they will not be "betrayed" by such a supervisor. Since voice behavior is often in as a risk action, we have reason to believe that subordinates have inclined to do it because they trust the one with whom they have close internal connection people. In other words, the close relationship supervisor-subordinates have achieved, the more likely for subordinates to make suggestions in front of their supervisors. Chinese culture especially emphasizes the social connection culture environment. Guanxi would play an essential role in deciding when and to whom the subordinates will proactively speak to. This social connection could operate as a protective mechanism for subordinates and a loyalty-inducing agent for supervisors to give a safe environment for voice behavior present [5]. Hence, the "guanxi" of the supervisor-subordinates would be the boundary effects we want to explore in this study.

Although research on the impact of leadership behavior discussions on subordinate advice has received attention from previous researchers, there is relatively little research considering the internal mechanisms of how supervisors influence their subordinates [6]. Moreover, there is a dearth of research on the public sector’s voice behavior psychological mechanisms. The PSM theory emphasizes that the more motivated employees are to serve, the more they are willing to work in public institutions and organizations; the more opportunities offered by government organizations for them to realize their potential to help others and society, the better they perform [7]. Previous studies have indicated that individuals with high PSM are more likely to exhibit positive attitudes and behaviors in the workplace, such as better job engagement, job satisfaction, job commitment, and organizational citizenship behaviors. Thus, this study will discuss the special motivation-public service motivation serve as a psychological mechanism by which leadership behavior influences the expression of voice behavior among subordinates, using the public service industry as an example.

As a special proactive behavior that aims to change organizations’ existing practices to improve their efficiency, the study of voice behavior has attracted much attention from researchers. Specifically, public sector employees also need to go beyond their formal job roles and engage in extra citizenship and proactive behaviors. Hence, this study will discuss the special psychology mechanism behind leadership behavior and voice behavior through public service motivation. Meanwhile, based on LMX theory, researchers emphasize whether the reliable relationship of supervisor-subordinates has been established would affect voice behavior. This study will also examine the guanxi’s boundary effects on affecting subordinates’ voice behavior. Therefore, we conducted a longitudinal survey with 136 administrative law enforcement officers of six public sectors in grassroots government to declare how and when these special administrative law protectors who worked in the public sector would like to voice. This study’s main conclusion provides a reference for guiding how to create an effective motivational environment in the public sector from a supervisor-subordinate relationship perspective.

## 2. Literature review and research hypothesis

### 2.1 Leadership-member exchange theory

Leader-member exchange (LMX) is an essential interpersonal relationship embodied in the workplace [8]. This theory’s core theory encompasses the ideas of role theory and social exchange theory. Role theory argues that leader-member exchange is a process of role-playing, internalization, solidification, and the role norms and obligations of the exchanging parties influence the exchange relationship’s direction [8]. Social exchange theory maintains that payoffs and feedback are used to preserve the exchange relationship and ensure its sustainability [9]. The essence of leader-member exchange is the feedback of the hierarchy embedded in the norm of reciprocity, while the quality of this feedback is different. Generally, a high-quality leader-member exchange involves the joint exchange of material and immaterial resources between superiors and subordinates [10], while low-quality leader-member exchange involves only an economic exchange between superiors and subordinates based on formal contracts [11]. If subordinates perceive a high-quality exchange relationship, they are more likely to perceive the supervisor as an "insider" [12].

In the relationship between superiors and subordinates in the public sector, we believe that there are both material exchanges between supervisors and members, as well as emotional exchanges such as a sense of support at work, and social exchanges based on the personal feelings of subordinates and superiors in non-work relationships. Since material exchanges between members of leadership in the public sector are fixed, there is no need to discuss them in terms of their relationships affecting individual behavioral performance. However, the non-material exchanges in the workplace and the personal exchanges based on personal feelings play a more critical and significant role in the willingness of subordinates to give advice. According to the basic idea of leadership exchange, the more subordinates are supported and affirmed by their superiors at work, the more subordinates are willing to reward their superiors with positive work achievements, and if the relationship between subordinates and superiors is at the same time very personal, with social ties outside of work, this makes the relationship between subordinates and superiors closer, i.e. subordinates are more willing to advise their superiors because they have a trust relationship with them It is also stronger in life.

Numerous scholarly studies have long emphasized that interpersonal relationships influence employee self-expression. For example, studies have observed that subordinates feel more psychologically secure when they perceive support and trust from their superiors, and the more they take proactive behavior to adjust and change their work behaviors [3, 13]. This suggests that many subordinates’ prosocial and proactive behaviors can be observed based on the exchange theory of leader-member relationships. But does the social connection affect the potential of the frequency of subordinates’ voice behavior is rarely discussed especially in the public sector? Thus, this study generates different subordinates’ voice expressions based on the idea that their behavior will be affected by their superiors’ exchange relationship.

### 2.2 Perceived supervisor support and employee voice behavior

Voice behavior was originally defined as a response to a perceived decrease in satisfaction with the work environment [14]. It is a bottom-up process in which individuals provide innovative changes and improvements to standard procedures [15]. Previous study finds that employees with higher felt obligation are more likely to participate through voice by expressing their positive concerns [16, 17]. Van Dyne and LePine (1998) [18] believed that voice is an important behavior in the workplace because it is an environment-improving, change-oriented, and constructive human-to-human communication behavior. Researchers have argued that voice behavior often reflects an employee’s inconsistent expression of inappropriate and unethical behavior toward a particular action within the organization, as well as an employee’s concern about an underlying organizational problem that has not surfaced or a willingness to share suggestions for improvement [17].

In recent years, researchers have conducted several extended studies on voice behavior all around the business management area, including the categorization of voice, behavior constructs based on internal motivation [19], direction [6], and content [20]. Besides, studies have explored the antecedents of voice behavior from personality traits and characteristics. Also, the organization factors are important at affecting employee’s voice behavior because whatever the form of worker organization, its function is to allow employees to participate, not necessarily through a formal setting but through all the rules and activities that directly affect their work lifes [21]. Employees participation such as voice to higher-up is a process in which influence is shared among individuals who are otherwise hierarchical unequals [22]. In a business simulation with students as participants that individuals high in a power hierarchy tend to voice their opinions to a greater extent than low positional power individuals [23].So participatory management practices balance the involvement of managers and their subordinates in information-processing, decision-making, or problem-solving endeavors [24].

Meanwhile, researchers have found the effects of voice behavior on beneficial organizational learning [25], enhancing employee job satisfaction, team innovation performance, etc. [26]. However, most of these studies considered voice behavior have been conducted in profit organizations, and the motor mechanism of voice behavior for public employees still needs to explore.While researchers have long emphasized employee voice behavior’s benefits, voices are often perceived as difficult tasks to advance [27]. Because of the potential threats and uncertainties associated with giving advice, sometimes it is not supported by supervisors or colleagues. Since supervisors in work situations are the ones with the power, resources, and influence to establish organizational norms and influence management practices [28], subordinates are more willing to engage in proactive behaviors when they have the support of the supervisor [29]. Based on the perspective of leadership member exchange, supervisors will shape members’ feelings of support for the possibility of self-realization and trust by empowering subordinates. It has also been demonstrated that the content, the size, and the support’s profitability will all promote the subordinates’ initiative when supervisors support their subordinates [27]. This suggests that in a work situation where the supervisor and subordinates are closely connected, a supportive social relationship between the supervisor and the subordinates will help subordinates develop a willingness to express themselves and feel as if they are "insiders." Consequently, subordinates are willing to provide suggestions for improvement based on the shortcomings of realistic work scenarios because their superiors’ support is the backbone of their safe voice. Since superiors’ support ensures that the subordinate has the initiative to challenge authority and is empowered and trusted to give advice, this supportive relationship allows the subordinate to express both types of promotive and prohibitive voices. This leads us to provide hypothesis 1.

**Hypothesis 1**: Supervisors support the positive effect of subordinate voice behavior on public employees.

### 2.3 Psychological mechanisms of supervisor support and subordinate voice behavior

Perry and Wise (1990) originally defined public service motivation as a "psychological deprivation or need" that people desire to eliminate or satisfy, referring to "an individual’s desire based on a personal tendency to respond to the motivations of public agencies and organizations." From another perspective, Vandenabeele (2007) [30] describes public service motivation as "a belief, value, and attitude beyond individual and sectoral interests, concerns the interests of a broader political organization, and motivates individuals to act accordingly when appropriate," which is the broadest definition of public service motivation. After nearly 30 years of research on the content, structure, and antecedent outcome mechanisms of public service motivation, it has become a more mature theory to explain public sector workers’ unique prosocial motivations. The theory has its origins in the western country while also receiving attention in the Chinese context. Researchers have revealed that a four-dimensional public service motivation measure is a stable and widely used tool in the Chinese context [31, 32].

This study proposes that public service motivation is a psychological mechanism by which subordinates are influenced by supervisor support and express voice behaviors for several reasons. First, according to the idea of supervisor-member exchange theory, supervisor support creates an environment in which subordinates perceive themselves as "insiders" and expresses the supervisor’s cooperation and empowerment to the subordinate’s behavior. Since the subordinate is also aware of his or her recognition by the superior, their motivation to perform public service will be strengthened by the subordinate’s protection and support in this exchange relationship. Voice behavior as an insider’s feedback on the feelings and results of their work will motivate them to provide service because they are inspired to do so. Besides, people with high public service motivation are more likely to be motivated in an environment that matches their public service intentions, and supervisor support indicates that supervisors expect their subordinates to do things that are good for the organization, in such a matching environment for high public service motivation individual, they willing to voice to meet the supervisor’s expectations and realize their career pursuit. As a result, they are more likely to provide a voice for development and point out organizational weaknesses. Supervisor support is a management style that allows and encourages freedom for subordinates, and in a psychological environment where subordinates are autonomous, competent, and satisfied with their relationships, individuals tend to have stronger motivations for conducting public service improving, and g things. Thus, voice behavior is more likely to occur when public service motivation is encouraged by the supervisor’s support environment. Hence, this study considers the hypothesis that public service motivation can act as a mediator between supervisor support and subordinate voice behavior.

The theory of public service motivation includes the rational motivation of interest in public policy-making and commitment to the public interest and the emotional motivation of self-sacrifice and compassion. Among them, the purposive motivation to serve the public interest while satisfying individual needs is a connotation of the motivation to be interested in public policy-making motivation; employees with strong policy-making motivation perceive working in the public sector as an opportunity to participate in public affairs, and the attraction to public political making as exciting and thrilling, reinforcing an image of personal self-importance [33]. From another perspective, commitment to the public interest is a norm-based motivation to serve, committed to the public interest, in which the individual aims to fulfill social responsibilities and norms [33]. Self-sacrifice motivation exists because individuals are willing to demonstrate their need for self-sacrifice by acting out of a desire to do good for others or the good of society. Such employees may have a greater willingness to serve society at the expense of their interests and preferences [7]. They are less likely to evaluate information and take action at their own cost because their values are highly associated with concern for the public [34]. Nevertheless, compassion represents a unique and distinctive emotional motivation, concern for emotional bonds formed with specific objects such as others, vulnerable people, disadvantaged groups, specific public, collectives, associations, and nations [35, 36]. The compassion that makes love and cares aims to allow for others’ protection and expresses "Empathy" and the reduction of agency motivation among public sector employees [37].

The rational part of public service motivation reflects the individual’s orientation to pursue public values, illustrating why they are willing to contribute to the organization’s future; its essence is that people seek to realize their identity as agents of public service. Thus, individuals with high agency motivation express a promotive voice as an initiative to participate in policy processes because they are concerned about the organization and social development. Also, agency motivation reflects a strong self-fulfilling need to serve in the public sector, provide public services, and satisfy one’s political pursuits. Additionally, a promotive voice is a creative expression of their desire to improve the organization without challenging existing authority or threatening their status. In a supportive leadership environment, subordinates are more likely to recognize the significance of their voice behavior and believe that their promotive voice can meet their organizational development goals and that the action is endorsed and supported by the supervisor.

Previous researchers have argued that Collectivism has caused a dichotomy in employee voice behavior in China. Specifically, the collective motivation to focus on the interests of the organization leads employees to offer their suggestions and ideas when identifying development dilemmas and the need for change, while the collective motivation to focus on harmony leads employees to keep their mouths shut and offer when identifying ineffective or outdated situations in the organization as a consequence of concern that pointing out problems will affect the relationship between superiors, subordinates, and colleagues emotionally motivated public sector employees, they are concerned with organizational interests. Empathy expresses their compassion for injustice and inequity, and self-sacrifice reflects their supreme willingness to give their all. These dimensions of public service motivation reflect a concern for the collective interest, putting devotion to society and the organization above self. Thus, motivated by this emotional motivation, individuals dare to point out perceived shortcomings in the organization because they are not concerned about their crisis due to the risk but are more worried about whether the organization can be improved. Consequently, they tend to make a prohibitive voice, helping their organization avoid unnecessary trouble. In a supportive leadership environment, individuals can achieve a high quality of quid pro quo relationships with their supervisors, that is, building common comradeships; meanwhile, they are convinced that challenging proactive behaviors are expected and expected by their supervisors and that it is the responsibility of "insider" to make prohibitive voice. Based on this reasoning, this study proposes a second hypothesis.

***Hypothesis 2***: *The public service motive acts as a mediator between supervisor support and voice behavior*.

### 2.4 The moderate effect of supervisor-subordinate’s guanxi (SSG)

In Chinese culture, the supervisor-subordinate’s guanxi is a concrete behavior; it played power over supervisory decisions on promotion and bonus allocation [38]. Some studies revealed that SSG could significantly affect sub subordinate’s voice behavior, such as career development, participatory management, and intentions to leave, as well as some proactive behaviors such as OCB and job crafting [39–42]. Moreover, SSG displays very small incremental validities beyond LMX theory in predicting outcomes in Miao et al.’s (2020) meta-analytic review [43]. The explanation about SSG impacting effectiveness at work lies in initiated outside of the workplace tie to link the supervisor and subordinates. A social resource is also provided in such exchange relationships and affects the subordinator’s performance positively [41]. Its concept regards that SSG “in China covers mainly non-work exchanges within the vertical dyad, and the benefits being exchanged can be social and economic in nature.”(Liu & Wang, 2013) [44], Although a great number of guanxi studies have confirmed its special positive effects on employee’s attitude and performance [5, 38], no empirical research to confirmed this tie in explaining the behavior of public employees, to our knowledge. In an environment like the public sector, power distance and bureaucratic hierarchy is the leading culture of people who worked in such an environment; public employees desperately need support and trust from their supervisor before action to decrease administrative risk and self-discretion mistake. Supervisor support creates an official link in the workplace to support their subordinates. Meanwhile, the SSG connection displayed a personal exchange and symbolized reliable informal support from the supervisor. By carrying these two parts of support insurance, subordinates would express their worries and ambitious voice, especially people who were internally motivated to perform something meaningful for society. Based on this analysis, this study proposes a third hypothesis:

***Hypothesis 3***: *The supervisor-subordinate’s guanxi would moderate the relationship between supervisor support and the subordinate’s voice behavior through the mediator of public service motivation*.

## 3. Research methods

To make sure all participants are willing to attend this survey and to get consent from the individuals who participated in our study, we informed the detailed information about this research and the two-time survey process, and showed all the information in the instruction part of the survey, if anyone is not willing to attend the survey, they could quit immediately. By asking respondents whether they would like to participate in the survey in the instructions of the questionnaire, we obtained their handwritten consent to participate in the study.

The study aimed at constituting a structural equation model to test the relationship of boundary effects of supervisor-subordinate guanxi (SSG) to supervisor support and subordinate voice behavior. Structural equation model is a multivariate technique combining aspects of factor analysis and multiple regression that allows an examination of the structure of relationships among the observed variables and latent variables and those among the latent variables. We used Mplus 7.4 software to conduct the structural equation model and investigate the pathway effects in this study.

### 3.1 Sample and research procedures

The empirical test was examined in a county in the middle province of China. All of the reporters in the study are grassroots administrative law enforcement officers, and they belong to 19 different county-level public administrative institutions. All public employees came from grassroots governments and public administration institutions. Before we start this survey, we already get a verbal commitment from the county’s main leader under investigation to avoid ethical problems. Participants in this survey were invited to participate in a survey at Time1 and Time2 six weeks later. In this survey, all the participants were invited to record their first letter of last names and last four telephone numbers, enabling individual responses to be matched over time by the research team. The present study was based on those participants who recorded their detailed information on both surveys. At the time1, 564 responses to the survey (representing a 77.47% response rate), the data of supervisor support, public service motivation, and supervisor subordinate’s guanxi were collected. Moreover, at time2, there were 291 responses to the survey (representing a 49.1% response rate), and the data on subordinates’ voice behavior was collected in time2. After deleting the cases with missing values, 136 employees came from 12 teams who completed both surveys and included detailed information at both times. Because of the huge unaverage number (minimal = 1, maximal = 17) of every group among 136 reporters, we defined the supervisor support at the individual level in the study. The final longitudinal sample of 136 employees was used in the study’s analysis.

### 3.2 Measures

#### Supervisor support

Supervisor support was assessed by the scale developed by Liu et al., (2010) [4]. We revisited these four items to investigate the supervisor’s support instead of the original scale used to identify the team’s support. Supervisor support asked respondents to describe how their felt about the antonymy support from their supervisors, such as “My supervisor supports the personal viewpoints of team members.” The response scale ranged from 1 (strongly disagree) to 6(strongly agree). The alpha coefficient for supervisor support in the study was 0.894. Because we conceptualized supervisor support as a shared feature

#### Voice behavior

We assessed voice behavior with six items developed by Van Dyne and LePine (1998) [18]. The specific item includes" I speak up and encourage others in this group to get involved in issues that affect the group.” The response scale ranged from 1 (strongly disagree) to 6(strongly agree). The alpha coefficient for supervisor support in the study was 0.904.

#### Supervisor subordinate guanxi

The supervisor subordinate’s guanxi was assessed with 5 items adapted from Chen and Peng (2008) [45] that asked participants to report their job-related positive supervisor subordinate’s relationship closeness, such as “My supervisor helped me solve work-related problems." The response scale ranged from 1 (strongly disagree) to 6(strongly agree). The alpha coefficient for supervisor support in the study was 0.924.

#### Public service motivation

We assessed public service motivation with five items from scales developed by Wright (2013). An illustrative item is “Meaningful public service is important for me.” The response scale ranged from 1 (strongly disagree) to 6(strongly agree). The alpha coefficient for supervisor support in the study was 0.846.

#### Control variables

We controlled the demographic variables such as age, sex, tenure, and education level in the study.

### 3.3 Measurement properties and sample differences

Correlations among all the scales for the longitudinal sample are listed in Table 1. Even though we collected the data two times, three variables, such as public service motivation, supervisor support, and guanxi were self-report at the same time. We first conducted a confirmatory factor analysis (CFA) of the 14 items from three scales at time1 to investigate the scales’ measurement properties. Meanwhile, we identified its common method deviation problems. This CFA test is based on the full sample of time1 rather than the longitudinal sample to use a larger number of participants. Three factors model fit indexes are *X*^*2*^ *= 416*.*589*, *df = 74*, *RMSEA = 0*.*07*, *CFI = 0*.*941*, *TLI = 0*.*921*, *SRMR = 0*.*042*. Correlations among the scales for the longitudinal sample were reported in Table 1.

**Table 1:** Means, standard deviations, and correlations.

Variables	M	SD	1	2	3	4	5	6	7	8
1. Age	39.31	8.74								
2. Sex	1.48	0.50	0.16							
3. Tenure	17.55	9.64	.91**	.24**						
4. Education Level	2.24	0.72	-.49**	-0.06	-.46**					
5. Supervisor Support	4.86	0.95	-0.09	-0.11	-0.12	0.04	(0.89)			
6. Guanxi	5.16	0.81	-.21*	-0.05	-.21*	.18*	.63**	(0.92)		
7. Public service motivation	5.05	0.88	-.20*	-0.08	-.27**	.27**	.50**	.43**	(0.85)	
8. Voice behavior	5.38	0.80	-0.05	-.32**	-0.10	-0.02	.33**	.20*	.21*	(0.90)

Notes: N = 136. Cronbach’s α appears along the diagonal in the brackets.

*p<0.05

**p<0.01 (two-tailed).

### 3.4 Results

**Fig 1:**
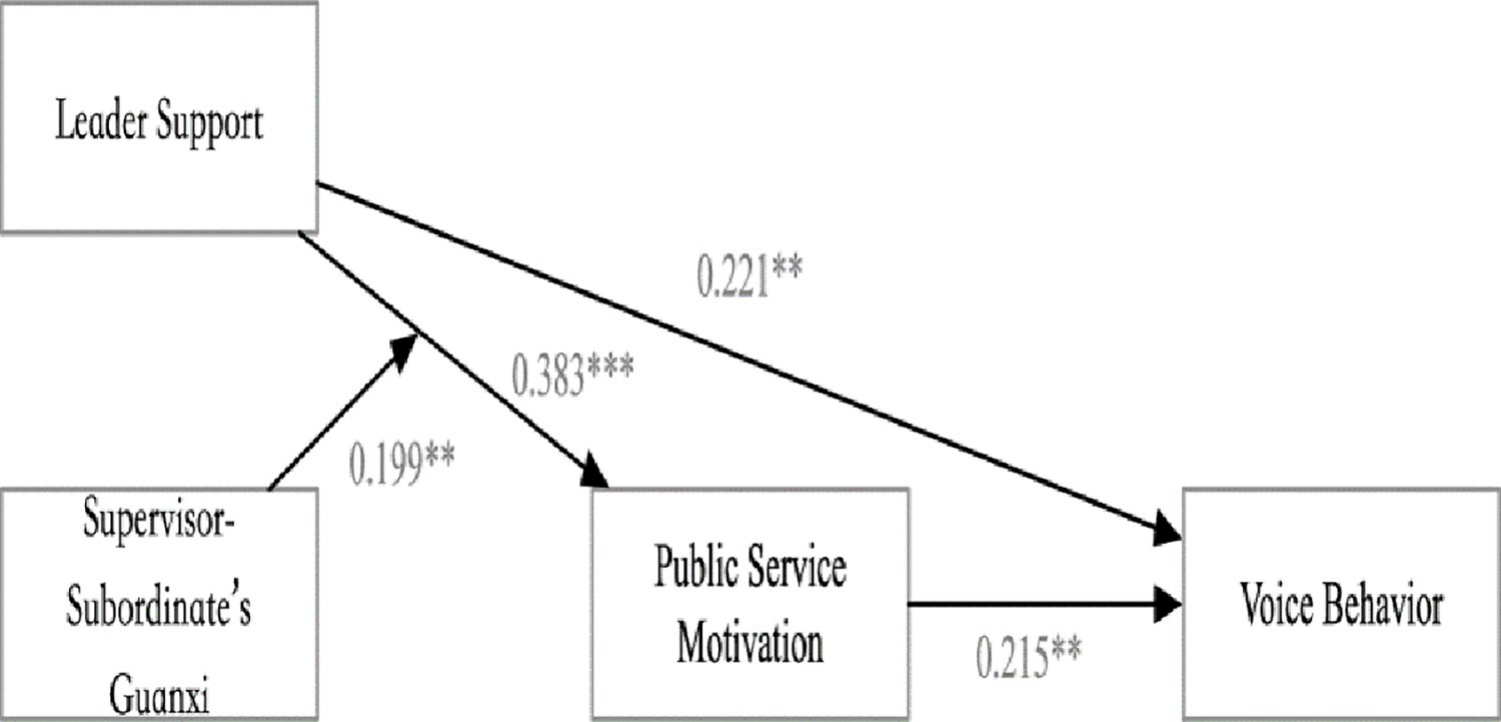
The interaction results of the study2.

**Fig 2:**
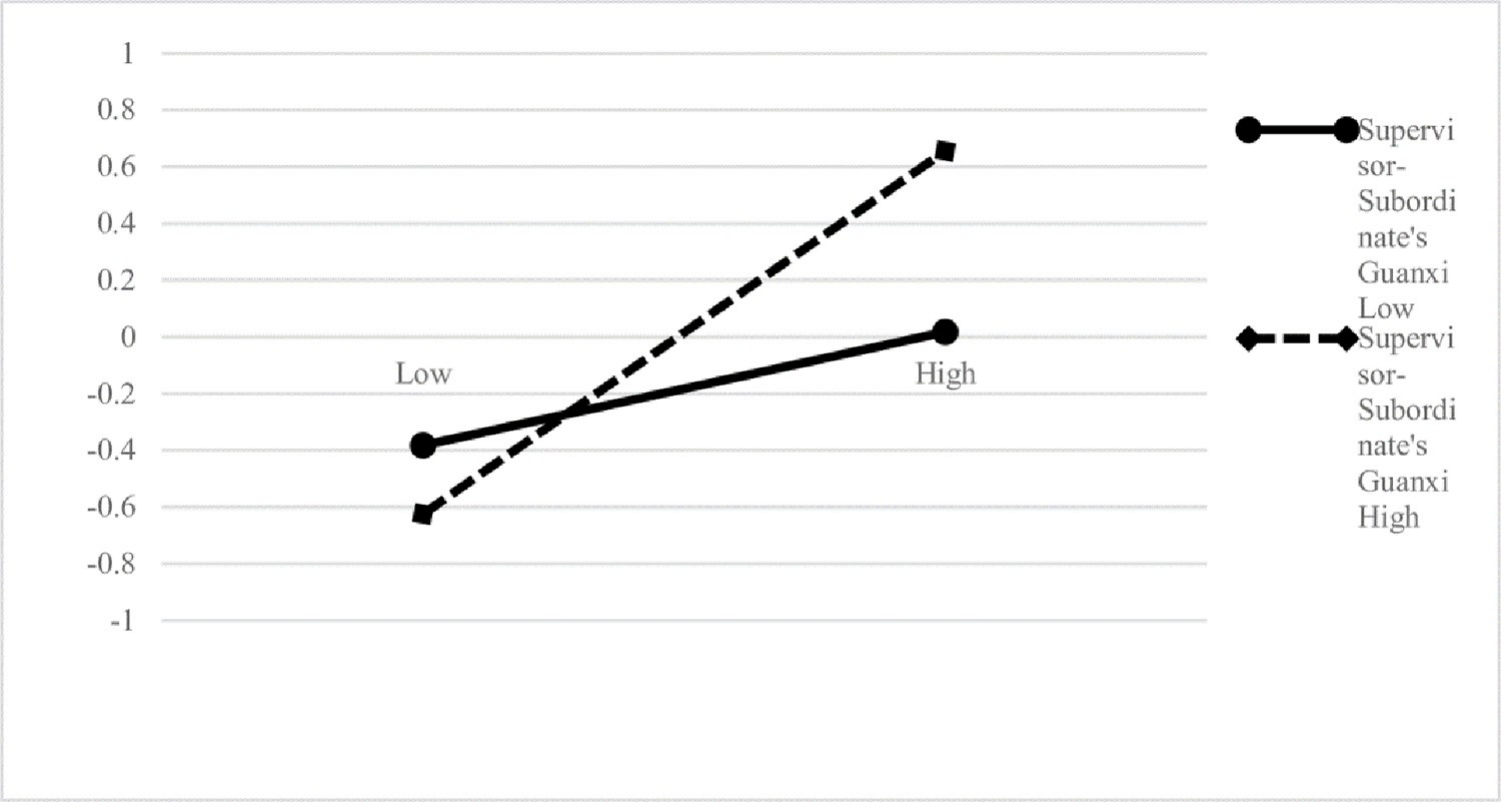
The moderating effect of supervisor-subordinate’s guanxi on the relationship between supervisor support and voice behavior.

To test the hypotheses, we assessed a moderated mediation model. The dependent variable was the Time2 measures of the superordinate’s voice behavior. Three other variables were the Time1 measures of supervisor-subordinate’s guanxi, supervisor support, and public service motivation. Moreover, we used the Bayes estimation to run 30000 times to obtain the regression results while the analysis by Mplus7.4. According to the results reflected in the table, after controlling all the control variables, the slope of the indirect pathway of supervisor support to voice behavior through the public service motivation is significant, from supervisor support to public service motivation is B = 0.383, SE = 0.1, p<0.001, 95% confidence interval is [0.174,0.567], hypothesis1 was verified. And the relationship between public service motivation to voice behavior is B = 0.215, SE = 0.098, p<0.01, and95% confidence interval is [0.018,0.401]. However, the direct effect is also significant now (B = 0.221, SE = 0.096, p<0.01), and the 95% confidence interval is [0.024,0.4], indicating that the public service motivation only played a partial mediator effect in explaining the relationship between supervisor support and voice behavior. Hypothesis2 was party supported. Moreover, the hypothesis proposed that supervisor support and supervisor-subordinates’ guanxi at Time1 interact to predict voice behavior at Time2. Table 2 illustrates that the proposed interaction was significant (B = 0.199, SE = 0.093, p<0.01), and 95% confidence interval is [0.009, 0.375]. The interaction is plotted in Figs 1 and 2.

**Table 2:** Indirect effects test of different conditions(bootstrap = 30000).

Level of Guanxi	Indirect effect	SE	95% confidence interval	
Low	0.28*	0.19	0.01	0.76
Median	0.33*	0.23	0.01	0.89
High	0.37*	0.27	0.00	1.02
Diff	0.09	0.07	-0.00	0.27

Notes: N = 136

*p<0.05.

As shown in Fig 2, the interaction pattern consists with our hypothesis, which declared that when Supervisor-Subordinate’s Guanxi was high(B = 0.37, p<0.05), the relationship between supervisor support and voice behavior was much more positively at the condition when Supervisor-Subordinate’s Guanxi was low(B = 0.28, p<0.05). Thus, our H5 was supported.

## 4. Conclusion

### 4.1 Theoretical contributions

In a high-speed changing world, the way governments innovate is by setting up a platform to encourage everyone to make suggestions, whether "praise" or "prohibitive" suggestions could be the source of vigorous government construction. The voices that came from insiders would undoubtedly help government adjustment and innovation, especially for our survey objects who worked in a frontline public sector who can acquire all sources of messages about promoting the work efficiently and protecting public interests instantly. Thus, it is vital to discuss how and when to motivate individuals to voice in the public sector.

Based on the above background, this study discussed how to create an environment that encourages insiders to contribute delightful and challenging suggestions in the public sector. According to the leadership-member exchange theory, this study reveals that a high-quality working relationship between supervisors and employees is the basis for inspiring subordinates to voices. Regardless of the voice’s contents, employees would like to speak because of this strong backing from the supervisors, and their voice behavior arousing is driven by rational and emotional public service motivation. Besides, outside the workplace, the social connection between the supervisor and the subordinate’s guanxi is also being considered in explaining the boundary effects of working relationships in explaining the subordinate’s voice behavior in our study. This finding showed the importance of informal connection in yielding subordinates’ positive behaviors. It gives us a holistic consideration about using the connection between supervisor and subordinates as a motivational tool in igniting enthusiasm in a public organization.

This study provides a psychological perspective on enriching proactive behavior in the public sector. This is worth trying because there is a lack of evidence motivating public employees to become proactive workers rather than passive ones in this challenging work context. This study provides a detailed psychological path based on the theoretical relationship between supervisors’ and subordinates’ formal and informal connection and their voice behavior. This would benefit researchers to understand proactive behavior more deeply since it opens a new micro-theoretical perspective for researchers to explore behavioral-oriented public administration issues.

Although the public service motivation theory has attained great attention in the public administration area, it desperately needs more exploring to find the effects of motivating an individual’s extra positive behavior. Previous studies have explored its effects in various positive performance aspects, but there is a lack of exploration of the behavioral predictions presented by non-role requirements and the boundary limits that exist for them, so there is a theoretical enhancement to further understand the effectiveness of public service motivation and its limits. Moreover, it is meaningful for the scholar to enrich the public service motivation itself and its complicated motivational effects.

### 4.2 Management insights

This study has the following managerial implications. First, it shows that supervisor support is a door opener for employees to voice in the hierarchical public sector, indicating the importance of supervisor and subordinate interactions in helping employees to develop their recognition and hence take the initiative at work. Second, it provided the idea that governments should emphasize individual initiative in today’s full of changing environments, considering that proactive individuals will help governments better identify management problems and reveal new methods to solve problems. Moreover, this study’s findings also confirm the importance of selecting employees with high public service motivation as the wise source for government to acquire innovation progress.

## 5. Limitations of the study

There are several shortcomings in this study. First, we used the structure equation model to test the relationship effects, but we settled the model in the individual level, and we haven’t calculated the cross-level difference. Since this study’s data were not evaluated to have significant team-level differences, we did not distinguish the individual and team-level differences when discussing perceive supervisor support. We could collect more data from different teams to be involved in future research. Although this study controlled for the effects of demographic characteristics which possibly affect voice behaviors, other variables such as employee attitudes and personality traits, job, task type, organizational characteristics, especially the public and private sector human resource management practices and cultural differences might also be the critical factor to influence employee voice behavior. Thus, these could be continued to be examined in the future. Also, our data all comes from one country, it might present the cross-country validity, so future study could further examine the relationship by a cross country design.
